# Adhesive intestinal obstruction increases the risk of intestinal perforation in peritoneal dialysis patients: a case report

**DOI:** 10.1186/s12882-018-0954-x

**Published:** 2018-06-28

**Authors:** Kentaro Fujii, Naoki Washida, Eri Arai, Masashi Tsuruta, Shu Wakino, Hiroshi Itoh

**Affiliations:** 1 Department of Internal Medicine, Keio University School of Medicine, 35 Shinanomachi, Shinjuku-ku, Tokyo, 160-8582 Japan; 2 Department of Nephrology, International University of Health and Welfare School of Medicine, 4-3 Kouzunomori, Narita, Chiba, 286-8686 Japan; 3 Department of Pathology, Keio University School of Medicine, 35 Shinanomachi, Shinjuku-ku, Tokyo, 160-8582 Japan; 4 Department of Surgery, Keio University School of Medicine, 35 Shinanomachi, Shinjuku-ku, Tokyo, 160-8582 Japan

**Keywords:** Bowel perforation, Peritoneal dialysis, Peritonitis, Adhesive intestinal obstruction

## Abstract

**Background:**

Peritonitis secondary to bowel perforation is a rare and potentially fatal complication in peritoneal dialysis (PD) patients. However, the early diagnosis of bowel perforation is difficult in PD patients because the initial symptoms and signs of bowel perforation are similar to those of PD-associated peritonitis. Furthermore, the risk of bowel perforation in PD patients is unclear. Here, we present a case of intestinal perforation located at the site of adhesive intestinal obstruction in a PD patient.

**Case presentation:**

A 73-year-old man on PD presented with progressive worsening of abdominal pain and cloudy peritoneal fluid. The peritoneal fluid cell count was increased to 980/ml and peritoneal dialysis-associated peritonitis was diagnosed. Computed tomography showed local adhesions causing agglomeration of the dilated intestine. He initially responded to antibiotic treatment; however, his abdominal pain was rapidly worsened after resumption of oral intake. On hospital day 23, computed tomography showed loss of contents from the dilated intestine and discharge of fecal material from the PD tube was noted. Thus, small bowel perforation was diagnosed, and he underwent ileocecal resection with colostomy creation. As indicators of EPS was not evident, PD catheter was removed. Since then, he has been on maintenance of hemodialysis since then.

**Conclusion:**

The findings of the present case suggest that adhesive intestinal obstruction in PD patients can increase the risk of intestinal perforation. Careful monitoring for the early detection of intestinal perforation is required in such cases.

## Background

Peritonitis secondary to bowel perforation is a serious complication with a high mortality rate of 46.3% [1]. Despite appropriate surgical procedure is required, the early diagnosis of intestinal perforation is difficult in peritoneal dialysis (PD) patients because the initial symptoms of intestinal perforation are similar to those of PD-associated peritonitis. In a previous report, intraperitoneal free air, which is a definitive sign of intestinal perforation, was found by Computed tomography (CT) in 30% of PD patients without intestinal perforation [2]. Thus, abdominal CT cannot be used as a diagnostic tool for the early identification of perforation peritonitis in PD patients.

The causes and risks of intestinal perforation in PD patients are unclear. Diverticulosis is known to cause large bowel perforation in PD and non-PD patients [3]. Approximately 20% of small intestinal perforation cases in PD patients are secondary to encapsulating peritoneal sclerosis (EPS) [4]; however, the causes in 50% of intestinal perforation cases are unknown.

Here, we present a case of small intestinal perforation located at the site of intestinal obstruction in a PD patient. An adhesive intestine caused by PD-associated peritonitis is uncommon; however, it can be a risk factor for intestinal perforation. Additionally, careful monitoring for the early detection of bowel perforation is considered important in such cases.

## Case presentation

**Fig. 1:**
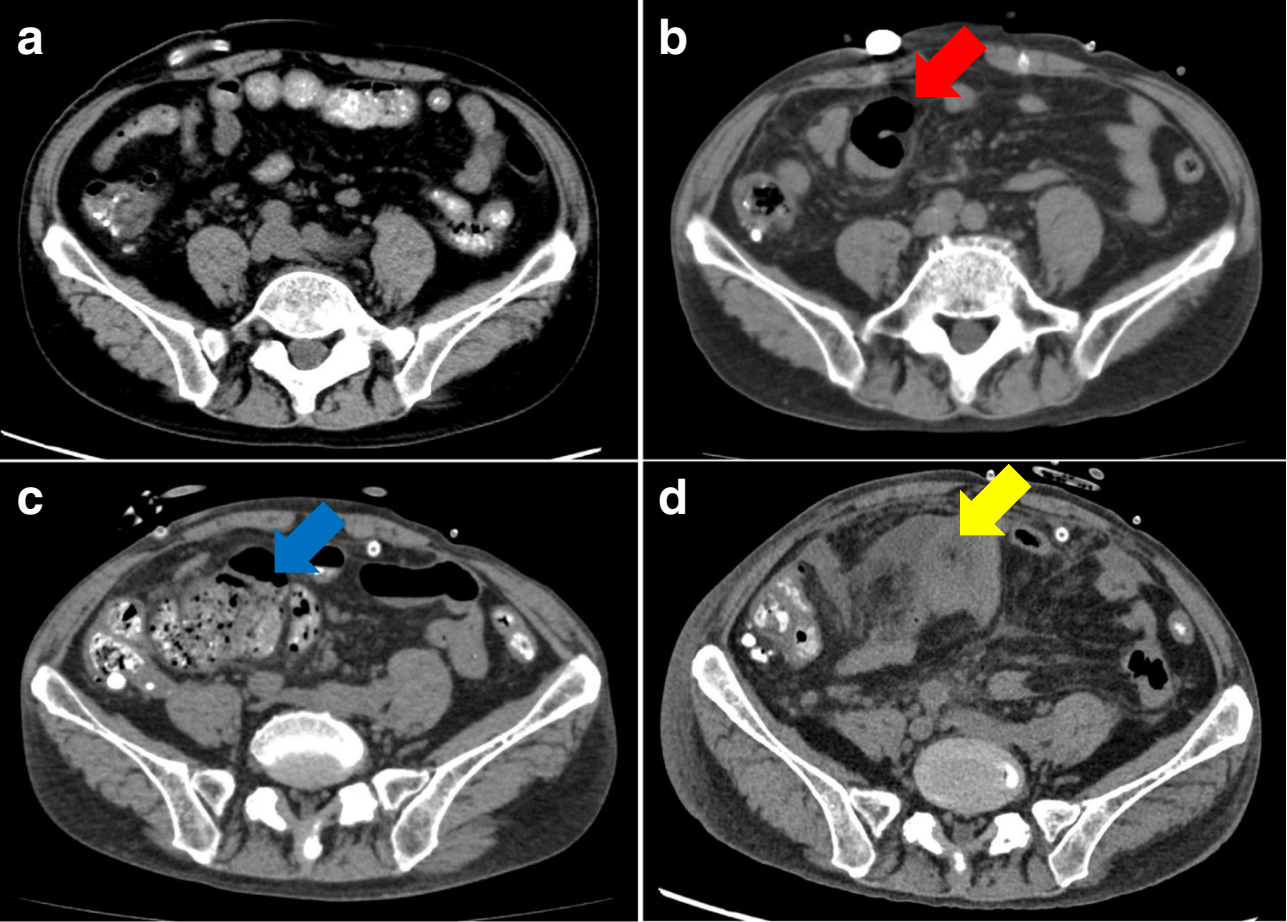
Longitudinal abdominal computed tomography images. **a** Bowel adhesion had not been noted 5 years previously. **b** Local adhesions encapsulating the intestine (red arrow) are detected after an episode of peritoneal dialysis-associated peritonitis 1 year previously. **c** Localized dilation of the intestine, suggesting adhesive bowel obstruction (blue arrow), is noted on admission. **d** On hospital day 23, it is seen that the intestinal contents disappeared and the dilated intestine collapsed (yellow arrow), indicating that the intestinal contents had leaked into the abdominal cavity

A 73-year-old Japanese man on PD presented with progressive worsening of abdominal pain and cloudy peritoneal fluid. He had high blood pressure, and he started continuous ambulatory peritoneal dialysis (CAPD) because of hypertensive nephrosclerosis 8 years previously. A PD catheter was primarily inserted at the right abdomen, but it was removed and inserted at the left abdomen because of exit site and tunnel infection 5 years previously. He had no past medical history of diabetes mellitus and major abdominal surgery. In the peritoneal equilibration test, his result was high. Bloody ascites was not evident. One year previously, he had been hospitalized for PD-associated peritonitis caused by touch contamination that was treated with intraperitoneal cephazoline and cephtazidime. Bowel adhesion was not noted 5 years previously; however, local bowel adhesions and agglomeration of the intestine were detected by computed tomography (CT) after the identification of PD-associated peritonitis (Fig. 1a, b). The major findings of EPS, such as peritoneal thickening and calcification, were not noted on CT.

On physical examination, his blood pressure was 134/74 mmHg, pulse rate was 76 beats/min, and temperature was 99.7 ° F. He complained of severe pain in the right upper quadrant of the abdomen, and this area was tender on palpation. The exit site was clear. Laboratory tests revealed mild inflammation, with a white blood cell count of 10,100 /μL and C-reactive protein level of 0.9 mg/dL. The peritoneal fluid cell count was increased at 980 /mL. Based on these findings, PD-associated peritonitis was diagnosed. CT showed localized dilation of the intestine, which suggested adhesive small bowel obstruction (Fig. 1c). As we suspected that the peritonitis might be associated with bacterial translocation from the dilated intestine, he was advised to stop eating and was switched from CAPD to hemodialysis. Additionally, he was treated with intravenous vancomycin and cephtazidime. The PD catheter was flushed once a day to prevent catheter obstruction with fibrin, and the characteristics of the peritoneal fluid were monitored. His abdominal pain was resolved and peritoneal fluid cell count decreased to < 30/mL, and thus, he resumed oral intake on day 8.

**Fig. 2:**
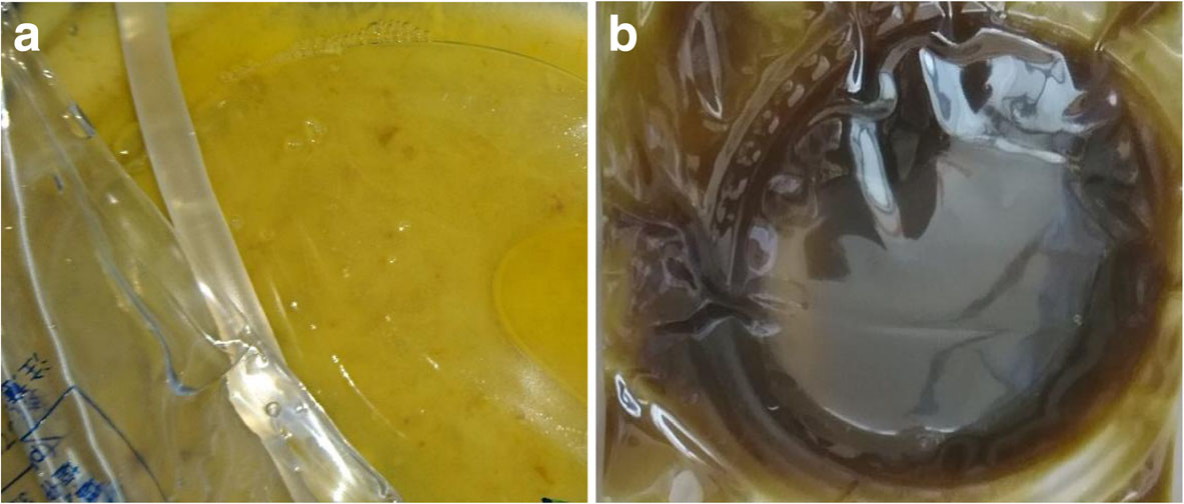
**a** After resumption of eating, the peritoneal fluid cell count shows a dramatic increase and the peritoneal fluid appears cloudy. **b** On day 23, fecal material with foul smell is identified from the peritoneal dialysis tube

**Fig. 3:**
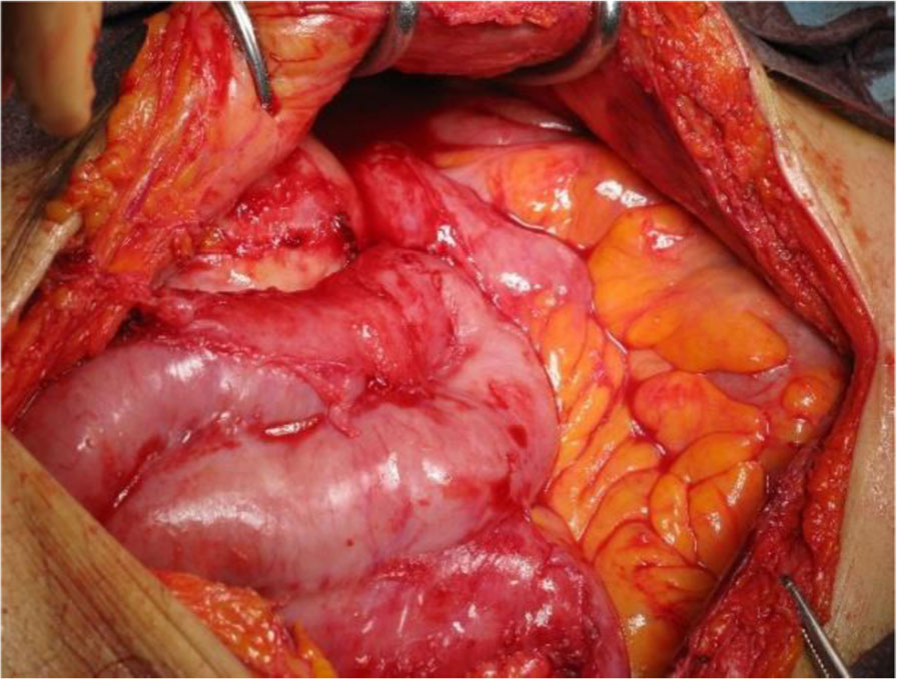
Although Intra-abdominal adhesions are severe, fibrinous encapsulation of the bowel, which suggested encapsulating peritoneal sclerosis, is not detected macroscopically during surgery

**Fig. 4:**
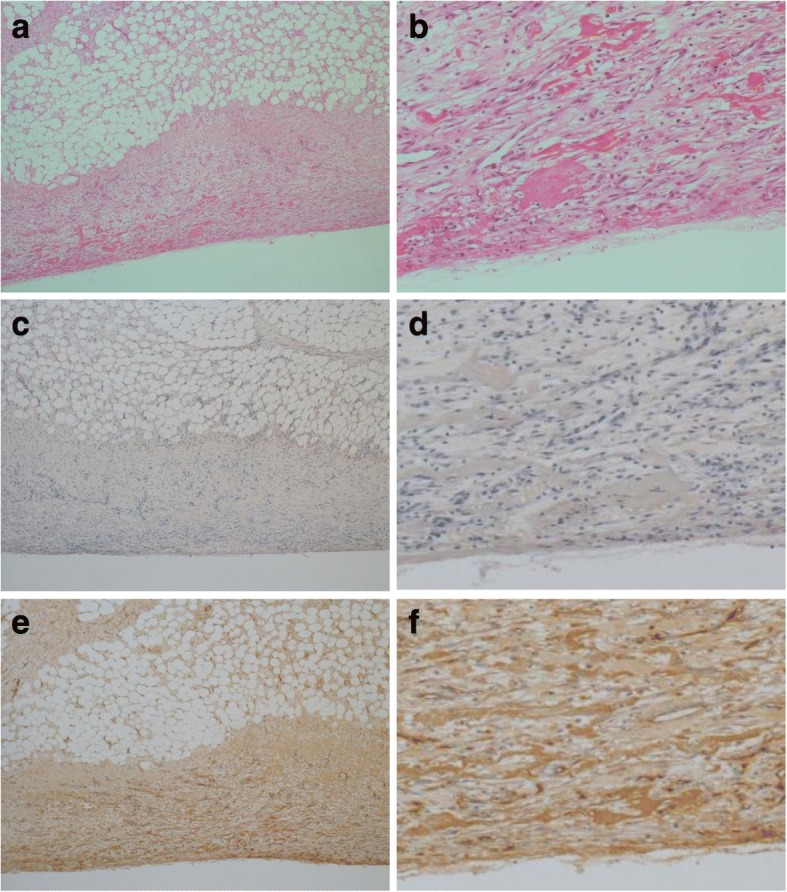
**a-b** Periodic acid-Schiff stainings of the resected intestine shows revealed inflammatory cells in the intestinal wall; however, marked intestinal fibrosis is was not evident pathologically. **c-d** Congo red-positive amyloid is not observed in the intestine. **e-f** Beta-2 Mmicroglobuline (B2M) immunohistochemistry shows only weak and non –-specific staining for B2M in the tissue fluid. These deposits did not indicate represent amyloidosis because Congo red staining was negative. Therefore, it was considered that deposition of B2M did not contribute to intestinal vulnerability or ischemia in this patient

After resumption of oral intake, his abdominal pain worsened and his peritoneal fluid cell count dramatically increased to 9600/mL on day 15. The peritoneal fluid became cloudy with a high amount of fibrin and white blood cells (Fig. 2a). Although he stopped eating again, his abdominal pain did not improve, and fecal material with foul smell was identified from the PD catheter on day 23 (Fig. 2b). Culture of peritoneal dialysate on admission was negative; however, culture of peritoneal dialysate on hospital day 23 was positive for *Enterococcus faecalis* and *Bacteroides caccae*. On CT, the intestinal contents disappeared and the dilated intestine collapsed, indicating that the intestinal contents had leaked into the abdominal cavity (Fig. 1d). Considering these facts, intestinal perforation was diagnosed, and he underwent ileocecal resection with colostomy creation. Although intra-abdominal adhesion was severe, fibrinous encapsulation of the bowel, which would suggest EPS, was not detected macroscopically during surgery (Fig. 3). As indicators of EPS were not evident, the PD catheter was removed. The perforation site was located at the adhesive intestine. The tip of the peritoneal catheter was located in Douglas’ pouch, and it did not injure the adhesive intestine. Pathological examination of the resected specimen revealed inflammatory cells associatet with the peritonitis in the intestinal wall. Intestinal fibrosis, arterial alteration, and tissue calcification were not evident pathologically (Fig. 4a, b). Although his serum beta-2 microglobulin (B2M) level was high (41.05 mg/L), amyloidosis and deposition of B2M were not observed (Fig. 4c-f). The postoperative course was uneventful and left arteriovenous fistula surgery was performed on day 42. Since then, he has been on maintenance hemodialysis with no recurrence of peritonitis.

## Conclusion

Although the early diagnosis of intestinal perforation is obviously important for a better outcome, definitive signs of intestinal perforation are often lacking in PD patients. The initial symptoms of intestinal perforation are often similar to PD-associated peritonitis. According to the ISPD guidelines, anaerobic growth in peritoneal fluid or polymicrobial peritonitis is frequently associated with intra-abdominal events requiring surgical attention; however culture is often false-negative [5–7]. Furthermore, recent evidence has suggested that surgical management is needed only in few patients [8]. Thus, understanding and clarifying the risks of intestinal perforation requiring surgical management in PD patients.

Diverticulosis is one of the most common cause of colonic perforation [3]; however, most causes of intestinal perforation in PD patients are unknown, except for conditions secondary to EPS [4]. EPS is rarely seen in patients with a long duration of PD therapy, and its major risk is highly associated with peritonitis episode [9, 10]. Thickening and fibrosis of the peritoneum in EPS patients can lead to the formation of a fibrous cocoon that encapsulate the bowel resulting in the intestinal bowel obstruction, intestinal ischemia, and intestinal perforation. The diagnosis of EPS with radiological tests is non-specific and difficult, and the diagnosis often requires confirmation by laparoscopy or laparotomy [11]. Although the pathophysiology of simple sclerosis and EPS in long-term PD patients are similar, thickening of the sub-mesothelial cell layer, inflammation, arterial alterations, and tissue calcification are significant findings in EPS patients [12]. In the present case, despite long duration of PD therapy and local adhesions of the intestine, which resembled a cocoon, the indicators of EPS were not evident pathologically. Additionally, intestinal amyloidosis associated with deposition of B2M in the intestinal tract makes the intestinal wall vulnerable and increases the risk of intestinal perforation in dialysis patients [13]. However, in our case, intestinal amyloidosis was not observed by pathological examination.

Intestinal perforation in the present case was secondary to adhesive bowel obstruction, which is generally a major complication of intraperitoneal surgery [14]. Bowel adhesions is often seen in EPS patients; however, the typical findings of EPS were lacking in the present case. Thus, the bowel adhesion was thought to be caused by a previous episode of PD-associated peritonitis. The pathophysiology of bowel adhesion characterized by local inflammation and fibrosis, was shown to be similar to that of bowel adhesion secondary to intraperitoneal surgery and bowel adhesion in the early stage of EPS [15]. However, the prevalence of bowel adhesion caused by PD-associated peritonitis has not been reported. Interestingly, adhesive obstruction itself rarely leads to intestinal perforation in non-PD patients, however, it appears to be a risk factor for bacterial translocation associated with intraluminal hypertension. PD-associated peritonitis caused by bacterial translocation can make intestinal wall vulnerable because of infiltration of inflammatory cells, resulting in an increase in the risk of intestinal perforation in PD patients.

In the present case, antibiotic therapy and fasting initially improved peritonitis, indicating that the patient’s peritonitis on admission was caused by bacterial translocation and not intestinal perforation. However, improvement of adhesive intestinal obstruction was insufficient and persistent bowel obstruction led to intestinal perforation when the intraluminal pressure increased after restarting oral food intake. In conclusion, the combination of adhesive intestinal obstruction and peritonitis in PD patients can increase the risk of intestinal perforation. Close monitoring for the early detection of intestinal perforation is required in such cases, and the timing of oral food intake resumption should be considered carefully.

## Abbreviations

B2M: Beta-2 microglobulin
CAPD: Continuous ambulatory peritoneal dialysis
CT: Computed tomography
EPS: Encapsulating peritoneal sclerosis
PD: Peritoneal dialysis
